# On-farm crop diversity, conservation, importance and value: a case study of landraces from Western Ghats of Karnataka, India

**DOI:** 10.1038/s41598-024-61428-1

**Published:** 2024-05-10

**Authors:** G. M. Puneeth, Ravi Gowthami, Ashvinkumar Katral, Kerekoppa Manjunatha Laxmisha, Ramesh Vasudeva, Gyanendra Pratap Singh, Sunil Archak

**Affiliations:** 1 Division of Plant Genetic Resources, ICAR - Indian Agricultural Research Institute, Pusa Campus, New Delhi, 110 012 India; 2 ICAR - National Bureau of Plant Genetic Resources, Pusa Campus, New Delhi, 110 012 India; 3 Division of Genetics, ICAR - Indian Agricultural Research Institute, Pusa Campus, New Delhi, 110 012 India; 4 College of Forestry, University of Agricultural Sciences, Dharwad, Sirsi, 581 401 India

**Keywords:** On-farm conservation, Diversity, Documentation, Landraces, Western Ghats, Natural variation in plants, Plant breeding

## Abstract

Landraces are important genetic resources that have a significant role in maintaining the long-term sustainability of traditional agro-ecosystems, food, nutrition, and livelihood security. In an effort to document landraces in the on-farm conservation context, Central Western Ghat region in India was surveyed. A total of 671 landraces belonging to 60 crops were recorded from 24 sites. The custodian farmers were found to conserve a variety of crops including vegetables, cereals and pulses, perennial fruits, spices, tuber and plantation crops. The survey indicated a difference in the prevalence of landraces across the sites. A significant difference with respect to the Shannon-diversity index, Gini-Simpson index, evenness, species richness, and abundance was observed among the different survey sites. Computation of a prevalence index indicated the need for immediate intervention in the form of collecting and ex situ conservation of landraces of some crops as a back-up to on-farm conservation. The study also identified the critical determinants of on-farm conservation, including (i) suitability to regional conditions, (ii) relevance in regional cuisine and local medicinal practices, (iii) cultural and traditional significance, and (iv) economic advantage. The information documented in this study is expected to promote the collection and conservation of landraces ex situ. The National Genebank housed at ICAR-NBPGR, New Delhi conserves around 550 accessions of landraces collected from the Central Western Ghats region surveyed in this report. Information collected from custodian farmers on specific uses will be helpful to enhance the utilization of these accessions.

## Introduction

Plant genetic resources (PGR) are the foundation for crop improvement and global food security^1^. The genetic diversity of crop plants has been maintained by farming communities by cultivating landraces. Landraces are variously known as heritage, heirloom or primitive cultivars or folk and farmers’ varieties^2^. Villa et al.^3^ defined landrace as “a dynamic population of a cultivated plant that has historical origin, distinct identity and lacks formal crop improvement, as well as often being genetically diverse, locally adapted and associated with traditional farming systems.” Due to the adaptive evolution, landraces constitute a reservoir of genes for nutritive value and tolerance to biotic and abiotic stresses^4^. Landraces play a significant role in maintaining the long-term stability of traditional agro-ecosystems.

Since landraces are lost due to genetic erosion^5,6^, genebanks around the world have captured their diversity in the form of 7.4 million germplasm accessions^7^. Although these ex situ collections have been providing the base material for crop improvement programs around the world, the material is no longer continuously adapting to changes in the environment, such as new races of pest or diseases, or major climatic changes. On the other hand, the population conserved on-farm continues to be dynamic in response to changes in local biotic and abiotic interactions as well as selection by custodian farmers thereby retaining its adaptation to the local environment and its distinguishing characteristics. In fact, landraces continue to exist “on farm” resulting in a traditional set up of “conservation by cultivation”. On-farm conservation has been defined as “the sustainable management of genetic diversity of locally developed traditional crop varieties, with associated wild and weedy species or forms, by farmers within traditional agricultural, horticultural or agri-silvicultural cultivation systems”^8^. Advantage of adaptive evolution offered by landraces, such as locally adapted alleles and allele complexes, exist only under on-farm conservation^9^.

Researchers have been endeavoring to document the on-farm conservation activities in many parts of the world^10–14^. However, insufficient documentation, inadequate transfer of ethnobotanical relevance from generation to generation, lack of interest among younger generations and inefficient policy intervention have led to and poor conservation and inadequate exploitation of landraces in plant breeding.

Western Ghats are a chain of mountains lying along the western coast of peninsular India for a length of 1600 km with an average elevation of 1500 m above mean sea level covering Gujarat, Maharashtra, Goa, Karnataka, Kerala and Tamil Nadu^15^. Western Ghats in India form one of the 34 biodiversity hotspots in the world^16^. As one of the four biodiversity hotspots in India, Western Ghats are home to 5000 angiosperm species, of which 34% are endemic^17^. The region is a primary center of origin for many crop species and houses a vast diversity of cultivated and wild crop plants. In addition to diverse flora and fauna, the Western Ghats are also native to diverse social, religious, cultural and linguistic groups. The crop biodiversity of the Western Ghats region has been documented previously by Asha et al.^18^; Gajanana et al.^19^ and Ramachandra et al.^20^.

Landraces should not be perceived merely as farmers’ cultivars that are reservoirs of useful traits. Landraces in an on-farm setup also include components of cultural landscapes and conservation agriculture and vistas to new market opportunities. Therefore, standalone inventorization of material becomes an inadequate exercise of documentation. On the other hand, documentation of the landraces in the on-farm conservation context provides insights into food-systems and sustainability. The present study was conducted in the context of the Central Western Ghats region of Karnataka state of India (i) to document the on-farm landrace diversity and conservation practices and (ii) to determine factors affecting on-farm conservation practices.

## Methodology

### Sampling methods and data collection

**Figure 1:**
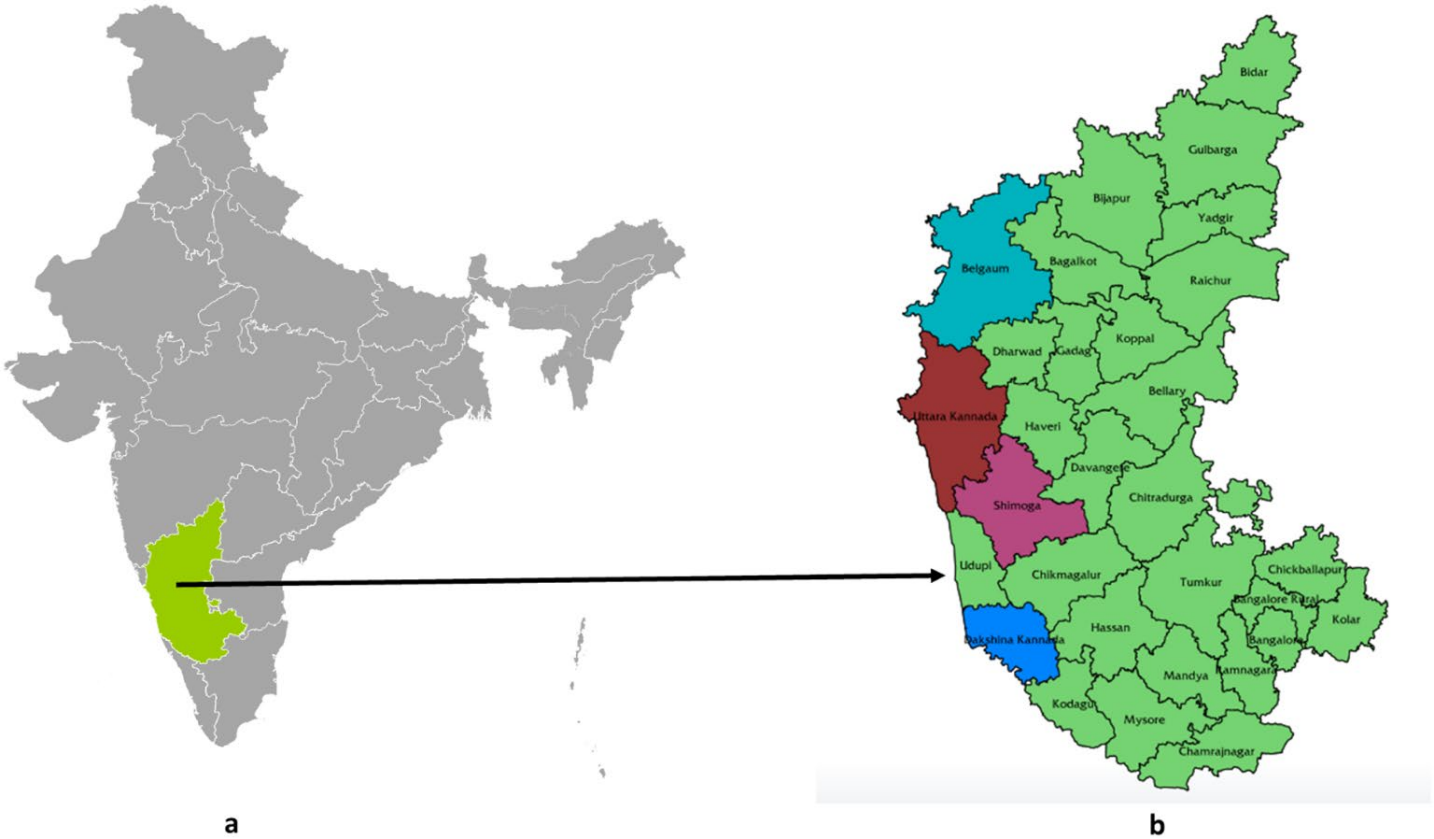
Map showing the survey area (**a**) India map highlighted with the survey state (**b**) Karnataka map with four survey districts (highlighted with different colours).

The survey to document the on-farm conservation was conducted in Central Western Ghats covering four districts viz*.,* Uttara Kannada, Shivamogga, Dakshina Kannada and Belagavi of Karnataka state in the southern part of India (Fig. 1). Initially, basic information regarding the distribution and type of crops, landraces and custodian farmers were collected by communicating with the resource-rich persons, local Krishi Vigyan Kendras (KVKs), agriculture colleges, non-government organizations and Protection of Plant Varieties and Farmers' Rights Authority, (PPV&FRA) New Delhi.

With the gradual replacement of landraces and changes in cropping pattern, there is a significant reduction in the on-farm conservation sites where landraces are cultivated. In such a scenario, where participants are difficult to locate, surveyors have used snowball sampling techniques^21^. We also used this nonprobability technique to identify potential on-farm conservation sites. Snowball sampling or chain referral sampling is a useful tool for analyzing rare instances or in our case, unexposed conservation sites. Resource persons from local KVKs and/or other organizations provided information about farmers conserving landraces in the respective jurisdiction.

**Figure 2:**
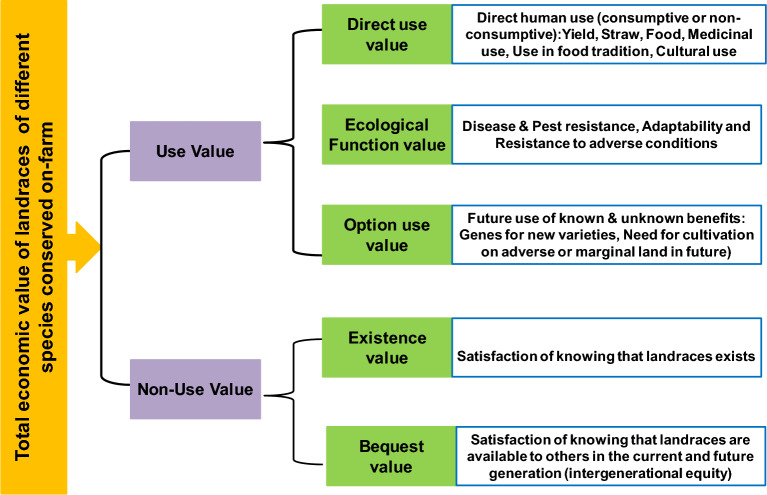
The nature of total economic value of landraces.

The survey was conducted from November 2020 to November 2021 to record the landrace diversity of different crops and their status. Based on the initial information obtained, farm households/farmers engaged in landrace conservation or mainly practicing low-input organic agriculture were pooled for information. The data was collected using socio-metric survey with snowball sampling technique^22^ where, custodian farmers were identified through data provided by other fellow farmers. A simple questionnaire was used to conduct the survey, which was based on an interview method and field observations. Farmers’ fields were visited to record the details of landraces and factors determining their conservation and use. Farmsteads were mapped based on geo-coordinates. Each landrace was recorded with botanical name, common name and the local name. Further, the farmers were also interviewed to obtain personal information, their farm details (landrace cultivation area, total farm area, cropping pattern), cultivation constraints, reasons for not growing landraces, community knowledge of biodiversity conservation and on-farm conservation, present and past use of landraces, traditional and cultural uses associated with the landraces, etc. Efforts were made to document the purpose of cultivating each crop species based on the total economic value (Fig. 2). In the entire exercise, we made sure to comply with relevant institutional, national, and international guidelines and legislations while documenting of on-farm conservation of landraces.

### Features of the study area

The Western Ghats Region in Karnataka, locally known as *Malenadu* lies between 12° 2′ 7″ N and 15° 44′ 46″ N latitude and 74° 14′ 3″ E and 75° 76′ 17″ E longitude. The Central Western Ghats Region extends through an area of 37,000 km^2^ covering areas of Chamarajanagara, Mysuru, Kodagu, Dakshina Kannada, Udupi, Hassan, Chikkamagaluru, Shivamogga, Uttara Kannada and Belagavi districts. The climate in the Western Ghats varies with the altitudinal gradation and distance from the Arabian Sea coast. The climate is humid and tropical in the lower reaches tempered by the proximity to the sea. Mean temperatures range from 20 °C (68°F) to 24 °C (75°F). The area is plain and the climate is humid along the west coast; thick forest and hilly area in the center; and tropical monsoon climate with undulated area towards the east side. Agricultural land in this region comprises of a variety of soil types including red soils, laterite soil, black soils and humid soils. Rice, spices, areca nut, jackfruit, cashew nut and sugarcane crops cover majority of the agricultural land area^23,24^. Weather parameters, land utilization and cropping pattern of the surveyed area are given in Table 1.

**Table 1:** Details of the surveyed districts in Central Western Ghats of Karnataka, India.

Parameters	Dakshina Kannada	Shivamogga	Uttara Kannada	Belagavi
Geographical area (km^2^)	4861	8478	10,291	13,433
Cropping pattern (%)				
Cereals	81.54	83.56	80.42	42
Pulses	4.81	0.83	1.99	6
Oilseeds	0.83	1.03	2.71	13
Commercial crops	0.09	7.30	7.92	33
Others	12.73	7.28	6.96	6
Land utilization (%)				
Forest	21.32	32.68	75.66	14
Net sown area	21.57	31.35	10.41	48
Other uncultivated area	34.17	24.37	7.31	12
Remaining area	22.94	11.60	6.62	26
Weather parameters				
Temperature(°C)				
(Max.)	36.6	37	37	38
(Min.)	26.1	23.2	16	12
Relative Humidity (%)				
(Max.)	90	89	89	88
(Min.)	70	64	64	40
Average rainfall (mm)	3524	1813	2887	844
Population (‘000)				
Rural	1094	1129	1018	3568
Urban	996	624	419	1211
Agriculturists (%)	2.38	12.42	9.98	14.79
Literacy rate (%)	83	80	77	73

### Statistical analysis

The data on the status of on-farm conservation and management in the region was gathered from multiple survey visits over a period of one-year. The data was analyzed by considering the cultivation of landraces across different districts in order to determine diversity and distribution. The following indices were computed to interpret the collected data:

$${\text{H}} = - \sum \left[ {({\text{Pi}}) \times \log ({\text{P}}_{{\text{i}}} )} \right]$$

where H = Shannon diversity index; P_i_ = Proportion of individuals of ith species in a whole community^25^.

$${\text{Pi}} = {\text{n}}/{\text{N}},$$

where n = Individuals of a given type/species; N = Total number of individuals in a community.

The Gini-Simpson index (or Simpson's index of diversity) measures the probability that the two randomly selected individuals belong to different species^26^.

$$(1 - {\text{D}}) = 1 - \frac{{n_{i} \left( {n_{i} - 1} \right)}}{{N\left( {N - 1} \right)}}$$

where *n*_*i*_ = Number of individuals in the *i*th species; and N = Total number of individuals in the community.

### Informed consent

The authors have obtained the consent from the custodian farmers and they are aware of the intended publication of information and images of the same.

## Results and discussion

### Landrace diversity

#### Inventory of landraces and prevalence

Landraces were documented from the Central Western Ghats spanning four districts of Karnataka. Snowball sampling allowed us to reach 24 farmsteads during the study. A total of 671 unique landraces belonging to 60 different crops were documented. These landraces belonged to fruits (8 crops, 181 landraces), vegetables (9 crops, 54 landraces), spices (8 crops, 40 landraces), pulse crops (4 crops, 15 landraces), plantation crops (3 crops, 11 landraces), tuber crops (4 crops, 32 landraces) and few miscellaneous plants (23 crops, 24 landraces). Rice was the only cereal crop documented in the region. However, out of the 671 unique landraces documented, 314 belonged to rice. Rice and fruits together accounted for three fourths of the total landraces documented.

The survey indicated that farmsteads with on-farm conservation and custodian farmers involved in cultivation of landraces were few and far between. It was clearly observed that even farmers immediately neighboring the on-farm farmsteads were not cultivating landraces. If a given landrace is cultivated at more than one on-farm conservation site, there is greater probability of continuity of conservation and availability. In other words, it is important to understand the prevalence of a landrace during the documentation process.

Based on the nomenclature synonymy of landraces, a Prevalence Index (PI) for on-farm conserved landraces was assessed as:

$$PI = \frac{{\mathop \sum \nolimits_{1}^{n} O}}{N}$$

where O is the occurrence frequency of landraces in the surveyed area; N is the number of landraces recorded; n is the number sites surveyed.

Out of 671 landraces documented during the survey, it was found that as many as 243 landraces were cultivated by two or more custodian farmers. It was observed that frequency of occurrence ranged from 1 to 7 (Table 2). The prevalence index was highest for banana (1.95). The PI of rice was 1.53 followed by chili (1.5), jackfruit (1.35), mango (1.33), ridge gourd (1.28), pepper (1.26), lab-lab bean (1.25) and brinjal (1.06) (Table 2). Meanwhile, for remaining 28 crops the value of PI was 1, showing that only one occurrence per landrace was recorded across the survey area.

**Table 2:** Prevalence of landraces in the survey area.

Crops	Landraces	Frequency of occurrence	Number of sites	Prevalence index
Banana	19	37	1–6	1.95
Citrus *spp*	9	9	1	1
Jackfruit	34	46	1–5	1.35
Jamun	6	6	1	1
Indian gooseberry	2	2	1	1
Kokum	6	6	1	1
Mango	104	138	1–5	1.33
Monkey jack	1	1	1	1
Ash gourd	3	3	1	1
Ladies finger	7	7	1	1
Bitter gourd	5	5	1	1
Bottle gourd	2	2	1	1
Brinjal	16	17	1–2	1.06
Chili	2	3	1–2	1.5
Cucumber	10	10	1	1
Red amaranth	2	2	1	1
Ridge gourd	7	9	1–2	1.28
Cardamom	4	4	1	1
Cinnamon	3	3	1	1
Clove	2	2	1	1
Coriander	1	1	1	1
Ginger	3	3	1	1
Nutmeg	2	2	1	1
Pepper	23	29	1–4	1.26
Turmeric	2	2	1	1
Arrow root	1	1	1	1
Sweet potato	5	5	1	1
Taro	8	8	1	1
Yam	18	18	1	1
Rice	314	482	1–7	1.53
Bengal gram	4	4	1	1
Cowpea	4	4	1	1
Horse gram	3	3	1	1
Lab-lab Bean	4	5	1–2	1.25
Arecanut	3	3	1	1
Betelvine	7	7	1	1
Coconut	1	1	1	1

A solitary farmstead practicing on-farm conservation of local landraces in a given area is matter of pride as well as concern. A family practicing on-farm cultivation of traditional cultivars singlehandedly contributes, as the custodians, towards conservation and perpetuity of landrace diversity. On the other hand, discontinuation of this practice by the custodian family due to any reason could possibly lead to irreversible loss of the landraces. Germplasm explorations by collectors may need to urgently focus on such landraces to ensure that these are conserved ex situ. Stakeholders may also look at promoting seed exchange among the farming community in order to increase their chance of “conservation by use”.

#### Importance of landraces

The landraces across farmsteads were documented to be conserved for their specific uses such as culinary purposes in raw, cooked, pickled and processed form, medicinal value or multiple uses (Table 3).

**Table 3:** Utility of landraces in the Central Western Ghats of Karnataka, India.

S. Nos.	Crop	Scientific name	Number of landraces	Uses of few landraces
	Fruits			
1	Banana	*Musa* sp.	19	Pseudostem used for kidney stones (*Betta baale*) Good for eat and chips, flowers as vegetable (*Deva Baale*) Medicinal value (*Kari Baale*) For vegetable curry preparation (*Palyad Baale*)
2	Citrus	*Citrus* sp.	9	For pickle preparation (*Huli Kanchikayi*, *Kanche Nimbe*)
3	Jackfruit	*Artocarpus heterophyllus* Lam	34	For paapad making (*Happala Sonte*) For making local food *kadubu* (*Kadubu Halasu*)
4	Jamun	*Syzygium cumini* (L.) Skeels	6	Tasty fruits (*Mandakki hannu, Kunnerale Kempu Meenangi, Hole Nerale, Bili Nerale*)
5	Indian gooseberry	*Phyllanthus emblica* L.	2	Pickle preparation (*Betta Nalli*)
6	Kokum	*Garcinia indica* (Thouars) Choisy	6	Mainly used as an alternative to tamarind to add sour taste in the foods In monsoon season, for *Huli* in sambar, oil from seeds (*Uppage Local*) Consumed as juice, food, and medicinal value (*Murugalu*)
7	Mango	*Mangifera indica* L.	104	For curry preparation (*Nekkare Maavu*) Pickling purpose (*Kengre Appe*, *Moodla Appe*, *Genasinakuni Jeerige*, *Vittala Jeerige Midi*)
8	Monkey jack	*Artocarpus lacucha* Roxb. ex Buch.-Ham	1	Used instead of tamarind, vinegar (*Whaate Huli*)
	Vegetables			
9	Ash gourd	*Benincasa hispida* Cogn	3	Edible fruits (*Kaashi Gumbala, Dodda Gumbala, Sanna Gumbala*)
10	Bitter gourd	*Momordica charantia* L.	5	For diabetes and as vegetable (*Sihi Haagala*)
11	Bottle gourd	*Lagenaria siceraria* (Molina) Standl	2	Used as vegetable (*Sihi Haagala*)
12	Brinjal	*Solanum melongena* L.	16	Bacterial wilt resistance and used for grafting (*Marabadane*) As vegetable
13	Chili	*Capsicum frutescens* L.	2	Medicinal value, pain killer, coolant, more spicy (*Sooji Menasu*)
14	Cucumber	*Cucumis sativus* L.	10	Mainly for juice preparation (*Ibbudla*), during summer season Bitter pulp mostly consumed by diabetes patients (*Oddu Southe*)
15	Ladies finger	*Abelmoschus esculentus* Moench	7	Used as vegetable (*Elale Bende, Kempu Bende, Sunkada Bende*),
16	Red amaranth	*Amaranthus cruentus* L.	2	In curry preparation Coolant (*Harive Soppu*)
17	Ridge gourd	*Luffa acutangula* Roxb	7	Used as vegetable (*Neera Peere*, *Pundi Heere*)
	Spices			
18	Cardamom	*Elettaria cardamomum* (L.) Maton	4	More yield (*Lambodi Thali*)
19	Cinnamon	*Cinnamomum verum* J. Presl	3	Culinary purpose (*Dalchini)*
20	Clove	*Syzygium aromaticum* (L.) Merr. and L. M. Perry	2	Highly fragrant and for culinary purpose (*Lavanga*)
21	Coriander	*Coriandrum sativum* L.	1	Culinary purpose (*Kaadu Kothambari*)
22	Ginger	*Zingiber officinale* Roscoe	3	Spicier and more pungent (*Kukku Shunti*)
23	Nutmeg	*Myristica fragrans* Houtt	2	Used for ayurvedic formulations (*Raama Patre*)
24	Pepper	*Piper nigrum* L.	23	More spicy, keeping quality is very good for 50–100 years (*Karimunda*)
25	Turmeric	*Curcuma longa* L.	2	More curcumin (7–8%) (*Jawari Arishina*)
	Tuber crops			
26	Arrow root	*Maranta arundinacea* L.	1	Boiled and consumed for its rich protein (*Kuvve Hittu*)
27	Sweet potato	*Ipomoea batatas* (L.) Lam	5	As vegetable (*Dhave Kanaga*, *Tambde Rattali*, *Thuppada Genasu*)
28	Taro	*Colocasia esculenta* (L.) Schott	8	Used for preparation of local food *patrode* (*Kunabi Mudli*, *Dhave Aalu*) Good for sambar preparation (*Dhaava Mudli, Cheduvale Mudli, Kaasaralu*) Green leaves in monsoon, immunity booster, healing of wounds as antiseptic (*Taikilo*)
29	Yam	*Dioscorea cordata* (L.) Raz	18	Leaves and tubers have medicinal value (vitamin rich) (*Chirike*)
	Cereals			
30	Rice	*Oryza sativa* L.	314	Puffed rice (*Bili Hegge*, *Mallige Sanna*), For making sweets and sweet dishes (*Bile Aloorsanna, Gulvadi Sanna, Indrani, Jeerige Saale, Kaagi Saale*) For dose and idli (*Chinnaponni, Mallige Sanna*) Red rice landraces to increase blood heamoglobin (*Hasudi, Hejje Batta*) Increases milk in lactating women, good for pregnant, more iron content (*Kare Gajuli*) and good for diabetes (*Rajamudi, Sorata*) Medicinal value for humans and livestock diseases (*Athikaraya, Chitaga, Kalave*)
	Pulses			
31	Bengal gram	*Cicer arietinum* L.	4	Used for culinary purpose (*Kempu Kadale, Hasiru Kadale*)
32	Cowpea	*Vigna unguiculata* (L.) Walp	4	Used for culinary purpose (*Kempu Halasande, Bannada Halasande*)
33	Horse gram	*Macrotyloma uniflorum* (Lam.) Verdc	3	Used for culinary purpose and good source of protein (*Kempu Huruli, Hasiru Huruli*)
34	Lab-lab Bean	*Lablab purpureus* (L.) Sweet	4	Used for culinary purpose and good source of protein (*Matti Avare*)
	Plantation crops			
35	Arecanut	*Areca catechu* L.	3	More yield and used in *paan* (*Dodda Adike*)
36	Betelvine	*Piper betle* L.	7	Good for *paan* (*Calcutta Yele*)
37	Coconut	*Cocos nucifera* L.	1	As tender coconut (*Tiptur Local*)
	Miscellaneous plants			
1	Basella	*Basella alba* L.	2	Coolant (*Kaadu Basale*)
2	Chebulic myrobalan	*Terminalia chebula* Retz	1	For making ink and have medicinal properties (*Alalekaayi*)
3	Common madder	*Rubia tinctorum* L.	1	Oil used for mental and nervous system ailments (*Jyothishnathi*)
4	Curry leaf	*Murraya koenigii* (L.) Spreng	1	Acts as coolant, reduces hairfall (*Karibevu*)
5	Galangal	*Alpinia galangal* (L.) Willd	1	Used in preparation of ayurvedic formulations
6	Illipe butter tree	*Madhuca longifolia* (L.) J.F.Macbr	1	Edible and medicinal properties (*Holesaalu Hippe*)
7	Indian sorrel	*Oxalis corniculata* L.	1	Immature fruits are edible and has medicinal properties (*Paappe Kaayi*)
8	Satawari	*Asparagus racemosus* Willd	1	Root used as lactogenic, nutritious
9	Snake fruit	*Elaeagnus conferta* Roxb	1	Decoction has medicinal properties (*Hulige Hannu*)
10	Bread fruit	*Artocarpus altilis* (Parkinson) Fosberg	1	Sweet fruits
11	Oblong leaf salacia	*Salacia oblonga* Wall	1	Edible fruits (*Balige Hannu*)
12	Pineapple	*Ananas comosus* (L.) Merr	1	Fruits have sweetness and are edible
13	Spanish cherry	*Mimusops elengi* L.	1	Edible fruits (*Ranjalannu/Salle Hannu*)
14	Star gooseberry	*Phyllanthus acidus* (L.) Skeels	1	Vitamin rich in leaves, medicinal value, used for curry and other local food preparation (*Nakshatra Nalli Kaayi*)
15	Malabar tamarind	*Garcinia gummi-gutta* (L.) N. Robson	1	Edible fruits (*Uppage*)
16	Indian coffee plum	*Flacourtia jangomas* (Lour.) Raeusch	1	Fruits are edible (*Mullu Sampige*)
17	Chow–chow	*Sechium edule* Sw	1	As vegetable (*Seeme Southe*)
18	Wild coriander	*Eryngium foetidum* L.	1	Culinary purpose, used in place of coriander (*Udda Kottambari*)
19	Kalmegh medicinal	*Andrographis paniculata* (Burm.f.) Nees	1	Used for fever (*Jeerad Kaddi*)
20	Drumstick	*Moringa oleifera* Lam.	1	Good source of minerals and vitamins (*Nugge Kaayi*)
21	Sugarcane	*Sachharum officinarum* L.	1	More sweet and juicy (*Kabbu*)
22	Bamboo	*Bambusa bambos* (L.) Voss	1	Tender bamboo to heat the body during monsoon (*Bidiru*)
23	Chicken weed/wild purslane	*Portulaca quadrifida* L.	1	Leafy vegetable, increase heamoglobin (*Golisoppu*)

#### Fruits

**Figure 3:**
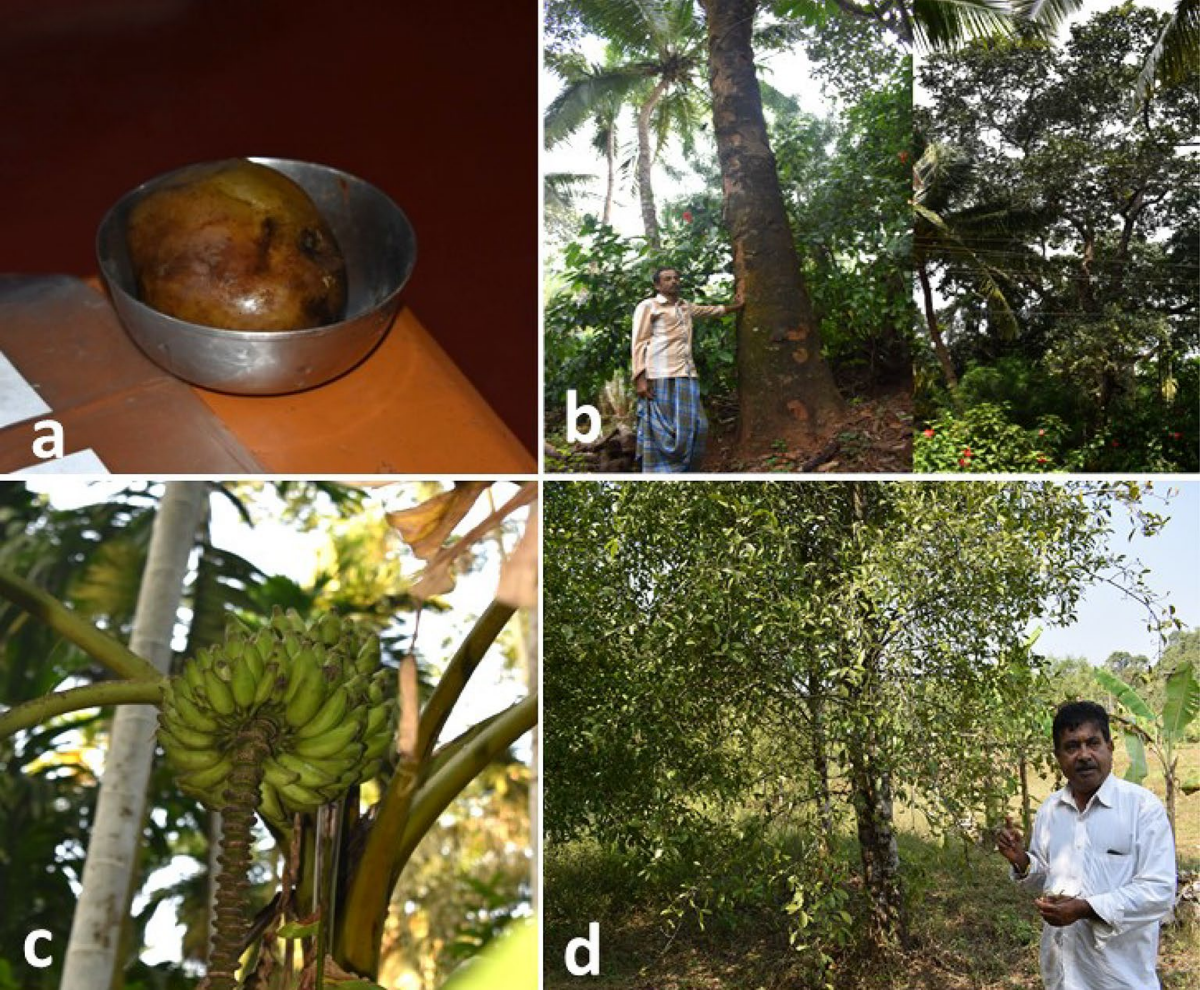
(**a**) *Neeru Kukku,* a local mango landrace, (**b**) farmer with original tree of ‘*Mankale Red’* a jackfruit variety, (**c**) *Elakki Baale,* grown in a farmer field, (**d**) A farmer with landrace, *Bili Murugalu*, of kokum.

Fruit landraces were found growing on the bunds, near the roadside, on marginal lands, in kitchen gardens and in between plantation crops in the fields mainly for personal use and not as commercial crops. Mango landraces have been conserved for their use in making pickles^27^. One of the unique landraces of mango to this region, *Appemidi* (used for pickle preparation only) has got Geographical Indication (GI) for Shivamogga and Uttara Kannada region. *Neeru Kukku,* a special landrace of Dakshina Kannada, can be soaked in salt water for 1–2 years without damaging the quality (named for this quality) and is also used in making *huli* for sambar (adds aroma and sour taste to sambar instead of tamarind) (Fig. 3a). This area also accommodates huge diversity of jackfruit, kokum and banana landraces than any region, owing for their adaptation and good growing conditions in the region. In jackfruit, soft fruited (*Biluva* or *Ambli* types) varieties are used in preparation of local cuisines (*idli, kadubu*) and hard fruited (*bakke* type) landraces are used in the production of processed food products such as *paapad* and chips (matured fruit before ripening); and is also consumed as a fruit. *Mankale Red***,** a landrace of jackfruit from Mankale, a place in Sagara (Shivamogga) has red fruits in which both rags and tendrils have a pleasant flavor when consumed (Fig. 3b). Banana landraces like *Elakki Baale*, *Mitli;* pineapple landrace like *Ananus Local* and jamun landraces are used as dessert fruit; *Hoo Baale* (flowers), *Kadhali/Deva Baale* (flowers), *Sakkare Baale* are used as vegetable (Fig. 3c). Landraces like *Betta Baale* (pseudo-stem is used for kidney stones) and *Kallu Baale* (used to treat kidney stones and grows on stones in the hilly regions) have medicinal properties. Meanwhile, some kokum landraces like *Uppage Local* (making *huli* for sambar, extract oil from seeds, juice preparation), *Muruga Huli* and *Punarpuli* (Fig. 3d) are well-maintained. Vasugi et al.^27^ assessed the diversity and morphological variations of *Appemidi* mango varieties in the same region. In the same way, Pradeep et al.^28^ observed cultivation of many landraces (> 20 each) in native crops such as mango and banana in Kerala, which is adjacent to our study area.

#### Spices

**Figure 4:**
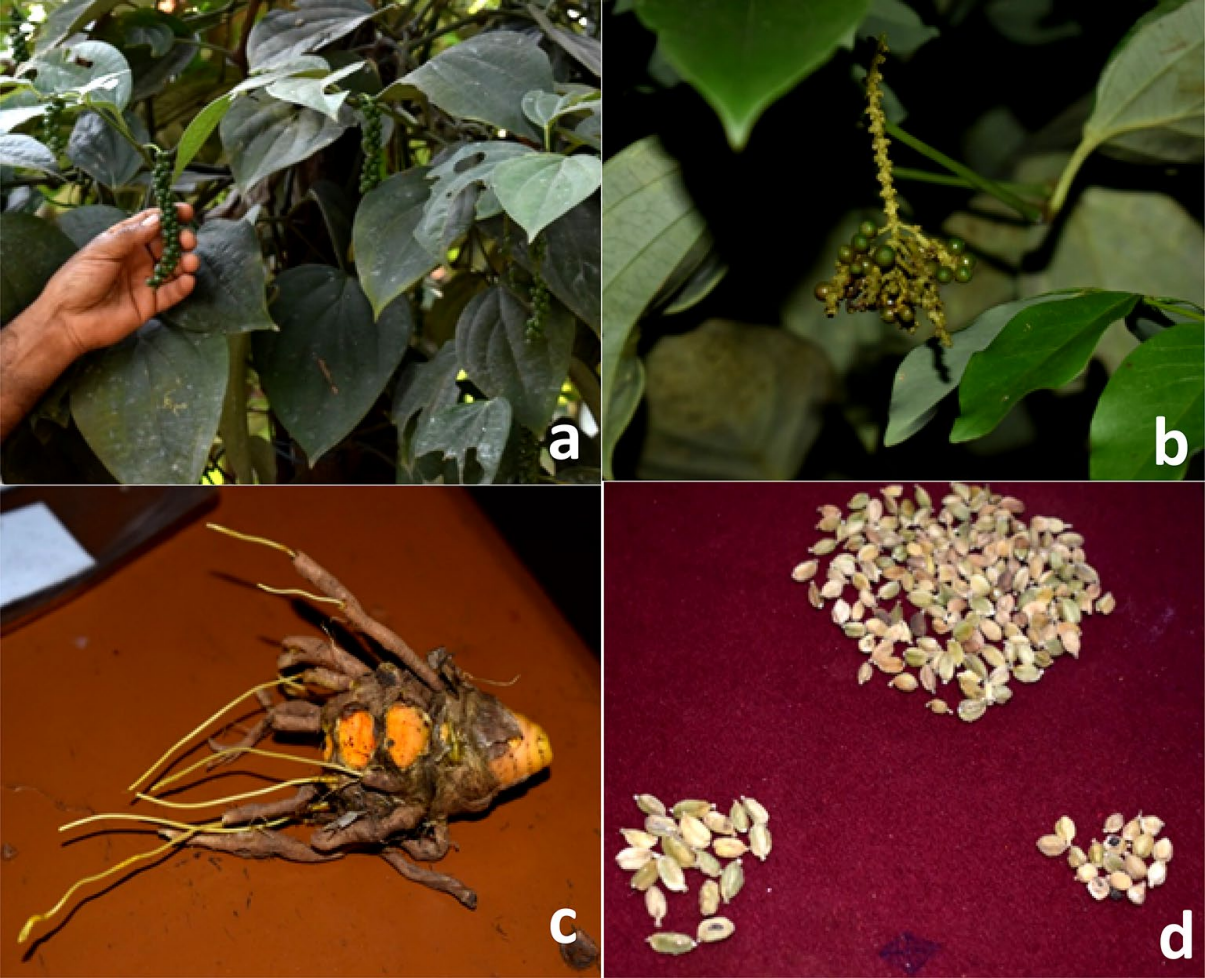
Representative photos of the spice landraces observed in the survey area (**a**) *Malligesara,* landrace of pepper, (**b**) *Thekkam Bunch Pepper*, landrace of pepper, (**c**) *Jawari Shunti,* landrace of turmeric (**d**) *Naati Yelakki,* landrace of cardamom.

Spices are high-value crops with large-scale export potential^29^. As a result, collecting and preserving spice crop germplasm is critical. In this regard, the Central Western Ghats holds a vast diversity of spice landraces for yield traits, resistance to disease and pests than improved varieties. *Karimunda* (spicier, good keeping quality i.e., 50–100 years), *Vakkalu*, *Gejje Hipli*, *Malligesara* (good yield potential) (Fig. 4a) and *Thekkam Bunch Pepper* (Fig. 4b) are few landraces of pepper^30^. *Maavina Kaayi Arishina* and *Kukku Shunti* were fragrant type landraces in turmeric and ginger respectively. While *Jawari Arishina* (turmeric) (Fig. 4c) contains more curcumin (7–8%) content and *Jawari Shunti* (ginger) is spicier and more pungent than the released varieties as per the farmers’ knowledge. Nutmeg used for preparation of ayurvedic formulations. Highly fragrant landrace of clove and some cardamom landraces like *Naati Yelakki* (Fig. 4d)*, Kilara, Lambodi Thali* and *Gundu Kaalu* were recorded during the survey. Saji et al.^31^ reviewed the conservation aspects and cultivar diversity of different spices of Western Ghats in particular and India in general.

#### Plantation crops

Plantation crops like areca nut, coffee, coconut and betel vine are the major crops grown in the region, hence their landraces too. Betel wine landraces namely, *Kasaravalli, Lakkavalli, Naagavalli* and *Panchavalli* are recorded during the study with limited information of the same regarding their importance and characteristics^31^. Areca nut landraces like *Sonda* and *Dodda Adike* are reported from the locality. *Tiptur Local* is a famous variety of coconut grown all over the Karnataka state.

#### Vegetables

**Figure 5:**
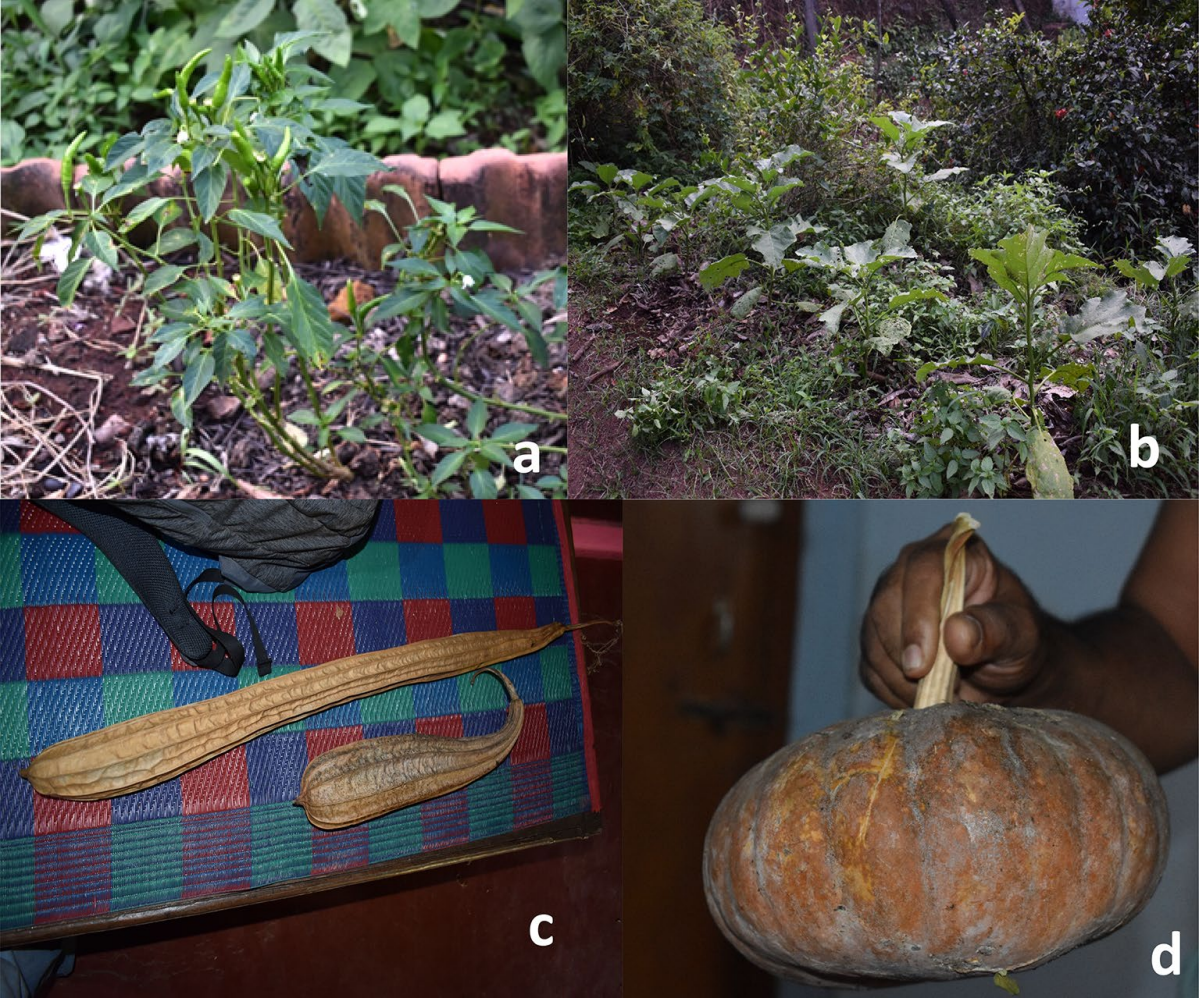
Representation of different landraces of vegetables (**a**) Chili, (**b**) Brinjal, (**c**) Ridge gourd, (**d**) Pumpkin.

Though the climate and weather of the Central Western Ghats region restrict the commercial cultivation of vegetables, many farmers grow landraces of different vegetables which are adapted to the locality’s soil and climate (Fig. 5). They grow, maintain, promote and preserve these landraces by harvesting the matured fruits for seeds to sow in the next generation. These landraces develop special traits over the years for its climate and soil conditions. Brinjal landrace *Udupi Mattu Gulla* has very thin skin and small spines on the fruit surface^32^. It has a unique taste and virtually gets dissolved while cooking and is also less astringent and less bitter when compared to other varieties of brinjal, and has got GI tag in the Udupi region, and *Marabadane/Kudane* (bacterial wilt resistance/used grafting) are among other brinjal landraces. Landraces of okra conserved are *Bahuvarshika Bende* (perennial), *Aane Kombu Bende* (very long fruits), *Sunkada Bende* (fruit contain protective hairs) and *Entugere Bende* (8 ridged fruit, large size) (Table 2). Cucumber is one of the important vegetables and many dual-purpose cucumber landraces are conserved. *Aane Mottu/Hegge Southe* (red pulp, pumpkin size, sweet taste), *Aati Southe* (in rainy season), *Ibbudla* (juice making), *Neeru Southe* (waterier content, grown near canals), *Oddu Southe* (for summer season, bitter pulp) are some of the landraces conserved on-farm. Landraces of other crops includes *Sihi Haagala* (bitter gourd) vegetable with no bitterness used by diabetes patients, *Sooji Menasu* (chili pepper) with spicy richness used for preparation of dishes and also act as a pain killer and a coolant. Likewise, Latha et al.^32^ recorded and documented different vegetables and their landrace diversity in the Western Ghats region of India. In a comparable manner the landrace variety of vegetables in the Italian Pugglia region was documented by Conversa et al.^11^.

#### Tubers

Tubers are important for food and nutrition security, as well as adaptation to climate change. Among the tuber crops, *Kunabi Mudli* (taro) (3ft long, big size, soft after boiling) is used in the preparation of *patrode* (a local dish). Most of the tuber landraces viz*., Bili Genasu, Thuppada Genasu, Kempu Genasu, Nagar Cone* and *Taambde Cone* (yam) are used as vegetable and making sambar. Some tubers like *Chirike* (highest vitamins) (yam) and *Taikilo* (taro) (immunity booster and healing of wounds as antiseptic) are also used for their medicinal properties. In a special case of *Kunabi* tribes in the Joida area of Uttara Kannada, different tuber crops and their wild relatives were documented. Some of the documented landraces are also mentioned by Asha et al.^18^. Similar documentation work in tuber crops was also conducted by Alwis et al.^10^ in Sri Lanka.

#### Cereals (Rice)

Rice is the staple crop of the region. Despite having many adversaries, many farmers are indulging themselves in cultivating, maintaining, promoting hundreds of rice landraces, which are very well adapted to the region’s climate and other agro-ecological factors. Some of the interviewed farmers are preserving hundreds of landraces because of their passion for conservation, market value, and in order to maintain the legacy of their ancestors. Some of the famous landraces maintained even now in the region are *Nereguruli Batta* (thrives in submergence for 40 days), *Rajamudi* (high tillering ability, organic cultivation, kernels are red and white rice type with soft rice, good for diabetes and was once patronized by kings of Mysuru Wodeyars), *Kayime* and *Kutti Kayime* (red seed kernel, high fodder yield, *rabi* season variety) and *Kempu Hasudi* (higher yield; resistant to diseases; red grains with good taste). Puffed rice (*Adnen Kelti*, *Bili Hegge*), medicinal value for humans and livestock diseases (*Athikaraya, Chitaga*), for making sweets (*Bile Aloorsanna*), for dose and idli (*Mallige Sanna*), aromatic rice and for making sweet dishes (*Gandasaale, Gulvadi Sanna, Indrani, Kaagi Saale*), red rice landraces to increase blood hemoglobin (*Hasudi, Hejje Batta*), can be grown in saline water (*Kagga Batta*), increases milk in lactating women, good for pregnant, more iron content (*Kare Gajuli*), good for snacks (*Mullare, Bili Halaga*), good for diabetes (*Rajamudi, Sorata*). Importance of rice landrace conservation and their characteristics are also highlighted by Rathi et al.^33^ in Chhattisgarh region and by Agnihotri et al.^34^ in Kumaon region of Uttarakhand.

#### Pulses

People consume pulses as their side dish along with staple food. There were six type of pulses were documented during the survey. Only few farmers are growing the pulses though not as main crop but as intercrop or in bunds. Lab–lab bean, a crop mainly grown in southern India, has different morphological variation in each landrace (*Chapparada Avare, Matti Avare, Katti Avare, Chaturbuja Avare*). Same for Bengal gram (*Kempu Kadale, Hasiru Kadale, Kappu Kadale*) and cowpea (*Kappu Halasande, Kempu Halasande, Bannada Halasande*) has variation in colour of the seeds and pods. Immature pods and leaves of some pulses use as vegetable.

#### Miscellaneous crops

India is known for traditional medicine system since ancient times. Thus, significance of the medicinal plants is known as part of Indian codified medicinal systems like Ayurveda as well as indigenous traditional knowledge about the medicinal uses by the community. In our survey, some plants were recorded for their multipurpose utility including medicine. The people in the region were found to treat various ailments since generations using local plants including Kalmegh (*Jeerad Kaddi*) for fever, Basella’s (*Basale Soppu*) leaves as coolant, wild purslane (*Golisoppu*) as leafy vegetable to increase hemoglobin, etc. Other popular plants included Malabar tamarind, Wild coriander, Indian coffee plum, Indian sorrel, Curry leaf, etc.

### Diversity indices

**Figure 6:**
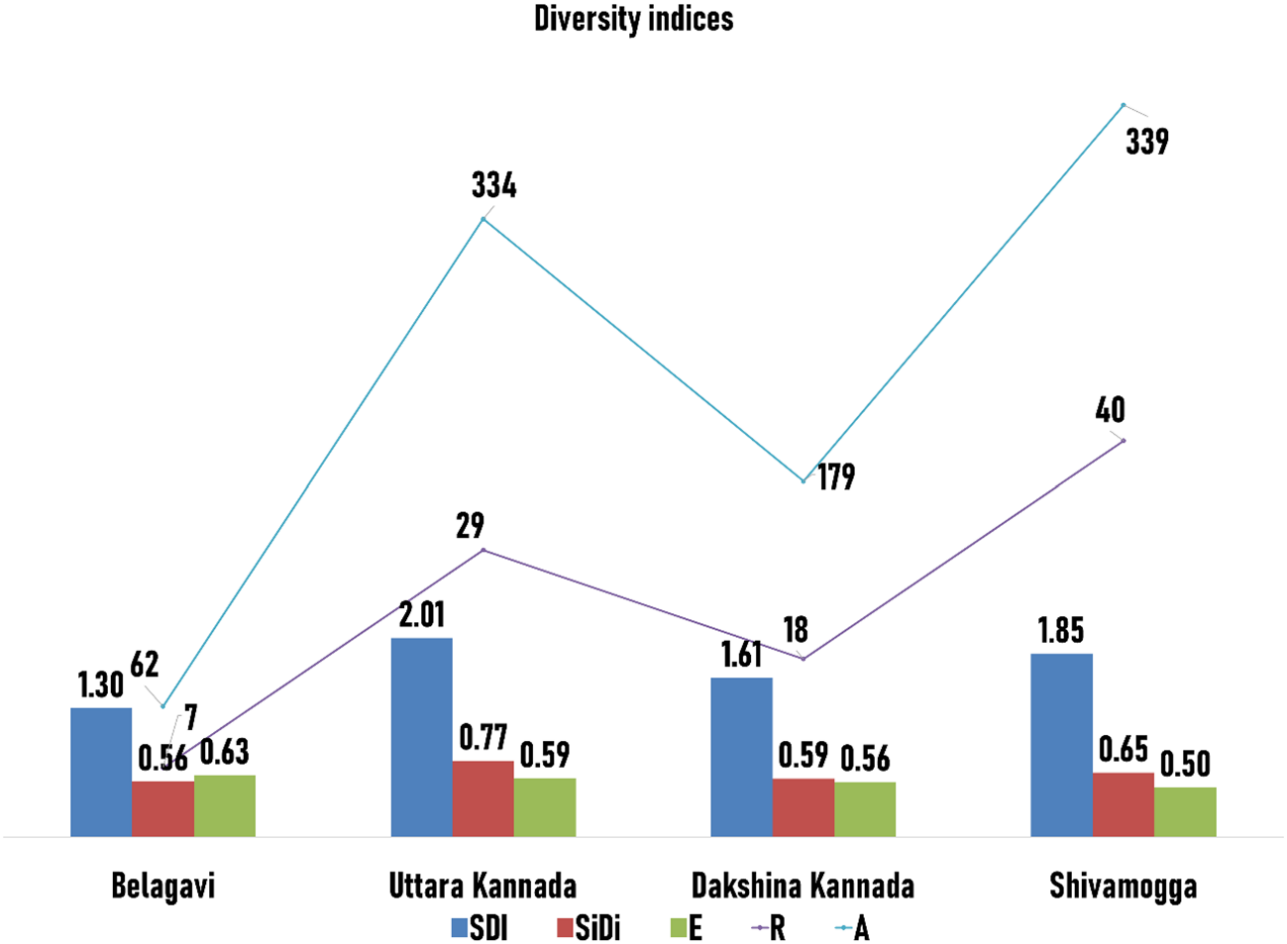
Diversity and distribution assessment of landraces using SDI = Shannon Diversity Index, SiDi = Gini-Simpson Index, E = Evenness. A = Abundance, R = Richness for four surveyed districts.

Based on the landrace nomenclature, Shannon-diversity index (H), Gini-Simpson index (1-D), Evenness (E), Species richness (R), and Abundance (A) were assessed between the crop groups of different districts. Significant differences in these parameters among the four study areas were observed. Shannon diversity index (H) dictates how diverse the species in a given area. Higher the index, more diverse the nature of species in that habitat^35^. Among the study areas, Shannon diversity (H) of Uttara Kannada (H = 2.01) was highest, followed by Shivamogga (H = 1.85), Dakshina Kannada (H = 1.61), and Belagavi (H = 1.3) had limited landrace diversity (Fig. 6). The value of Gini-Simpson's index (1-D) reflects how many different types of species are in a community and how evenly each species is distributed. Similarly, Uttara Kannada showed more diversity in landraces with a Gini-Simpson index value of 0.77 and Belagavi showed very less diversity with a value of 0.56. In terms Gini-Simpson index, Belagavi and Dakshina Kannada exhibited comparable diversity levels with values of 0.56 and 0.59, respectively (Fig. 6). Ocimati et al.^15^ assessed the same for *Musa* cultivars in Rwanda and found a lower diversity index, which was prone to genetic erosion. Species evenness (E) is the measurement of the relative abundance of different species. The species evenness ranges from zero to one, with zero signifying no evenness and one signifying complete evenness. Shivamogga had more diverse landraces than other districts in terms of Evenness (E) with a value of 0.5 followed by Dakshina Kannada (E = 0.556), Uttara Kannada (E = 0.591) and Belagavi (E = 0.625) (Fig. 6). The highest species richness (R) was observed in Shivamogga (40) followed by Uttara Kannada (29), Dakshina Kannada (18) and Belagavi (7) (Fig. 6). The current study revealed that Uttara Kannada and Shivamogga had more landrace diversity for their practice of sustenance farming in remote areas, use of landraces in local food systems, traditional and cultural links. While, Belagavi had less diversity in all the terms due to various probable reasons like commercialization of agriculture, use of more improved and hybrid varieties and, so on.

### On-farm conservation and management

#### Socio-economic characteristics of custodian farmers

Among the 24 sites of on-farm conservation, three farmers possessed more than 10 ha farm land, 12 had medium sized farms (2–10 ha), whereas nine were found to have small farms (< 2 ha). Landraces of field crops (rice and pulses) along with vegetables and tuber crops were found conserved mainly on the small farms. On the other hand, perennial species including fruits, spices and plantation crops were in medium to large farms. Among the 24 on-farm conservation sites, the age of the custodian farmers ranged from 35 to 75 years; majority (12) were in the age bracket of 40–60 years. The two young farmers (< 40 years) actively engaged in on-farm conservation were exclusively involved in commercially attractive activity of maintaining the rootstocks of traditional cultivars of perennial crops (pepper, mango and jackfruit) popular for their adaptation and resistance to pests and diseases^36^.

However, it was starkly clear in our survey that younger generation of farmers is not inclined to engage in on-farm conservation. It was evident from the interaction with farmers that traditional knowledge accumulated over the years with the experienced farmers could be in danger of not finding next-generation custodians.

From the current study, it was also found that majority of farmers are conserving many landraces dedicated to few crops instead of single landrace covering entire farming area. Few farmers conserve rice landraces, by growing most of the landraces in a 10 m^2^ area in order to maintain and preserve their self-interest and passion for conservation; and only a few landraces are grown in a large area because of their potential use. For example, *Nereguruli Batta* (rice) tolerates submergence condition up to 40 days in Shivamogga district. Fruits were grown majorly as an intercrop with spice and plantation crops. Some vegetables, fruits (mango and jackfruit) and miscellaneous crops were well-looked-after and maintained in home gardens for their use in preparing traditional dishes (mango-pickle, jackfruit-chips, *idli*) and traditional medicine. The marketing of the farm produce is distinct for different farmers based on the reason for cultivation. Many farmers grow landraces mainly for their personal use and are part of local food systems (pickling varieties of mango and vegetable landraces). Few landraces have cultural and traditional importance along with some quality traits that enjoy demand in local markets and weekly fairs (jackfruit varieties, local tuber crops). Very few landraces have demand in the countrywide market for their nutritional and medicinal importance (*Navara* and *Ambe Mohar* in rice).

Custodian farmers in the study area are majorly residing in remote villages and villages located in the vicinity of the forest. It was found that, the nearest proper road was 20–25 km away from the on-farm conservation sites. In the absence of market attraction, these farmers were found to cultivate landraces mainly for home consumption. At best, grains are sold at the local weekly bazaars and seeds are exchanged with fellow farmers and relatives. In exceptional cases, farmers who reside nearer to markets of nearby towns (< 10 km) were observed to get good prize for their produce.

Farmers generally designate local landraces names after specific characteristics. The appearance of seed and kernel, crop plants, taste, aroma, maturity, plant size, use and growing conditions are all crucial factors in determining a landrace name^33,37^. The same pattern of use/characteristics and other features are used to name traditional landraces/cultivars in the Central Western Ghats. It was observed that number of farmsteads having on-farm conservation activity was very less. Farmers belonging to post-green revolution era (born after 1970’s), tend to cultivate high yielding modern varieties with a focus on enhanced income generation. In the absence of formal documentation, the only source of information about names of landraces and their specialty uses is the senior farmers belonging to age bracket of 60 and above.

#### Determinants of on-farm conservation

Farmers have indulged in selecting, growing and maintaining landrace biodiversity within and among the crops in their fields and community seed banks from generation to generation. Farmers were well aware of the benefits of local cultivars, which includes high market value^10^, adaptation to adverse weather conditions, good eating quality, lodging resistance, resistance to pests and diseases, low production costs, and a consistent yield^14^. Scientific investigations in rice provided some insights into the utility of the landraces in crop improvement programs^38,39^. The Central Western Ghats is a partial forest area and few farms under this study are located in the vicinity of the forest area (Joida, Sagara, Sirsi, and Thirthahalli). The region receives heavy rainfall during monsoon (June–September) and a good amount of groundwater facility enhances agriculture in the region. Agriculture practiced in the region is mainly rainfed with few exceptions. This has led farmers to follow organic farming with fewer inputs, which indirectly chooses the local traditional varieties for their adaptation to the local environment for generations^14^.

From the present survey, it was observed that one set of farmers conserved landraces on-farm with sound knowledge on importance of landrace and conservation (direct conservation), while another set of farmers conserved landraces for their food and other needs without any knowledge on importance of landrace and awareness of conservation (indirect conservation). Special mention for the *Kunabi* tribes from the Joida area of Uttara Kannada, for their cultivation of unique rice and tuber crops’ landraces in marginal land, forest land and kitchen gardens for the sake of family sustenance and tradition without knowing the actual importance of these landraces in national plant genetic resources system. The distribution of tuber crop landraces in the area follows the ideal environment for their growth and development, such as soils, precipitation, elevations and drainage are in line with the results reported by Alwis et al.^10^ in Sri Lanka. This shows that many farmers were not aware of the concept of biodiversity conservation and on-farm germplasm conservation. However, they have contributed to on-farm germplasm conservation without their theoretical knowledge and awareness of germplasm conservation.

Though the economic benefit is the major driver of conservation^12,40^, non-economic factors like prestige for being the owner of diversity^12^, exchange of specific landraces and their products with neighbors, relatives and family friends^22^ are among the others which motivate farmers to engage in on-farm conservation. Landraces/traditional folk varieties are also conserved because of their adaptability to agro-climatic conditions viz., higher rainfall in the western side of the Western Ghats (adaptation of rice landraces for rainfed condition (*Kayime*), *Rabi* season (*Kutti Kayime*) in Dakshina Kannada, low fertility of forest soils in Uttara Kannada (tuber crops in Joida)^18^ and Shivamogga. Socio-economic conditions including fragmented land, limited availability of inputs, poor financial condition of farmers and tolerance to biotic and abiotic stresses (e.g., *Anthara Saale* for drought and weed tolerance in rice; *Marabadane/Kudane* for bacterial wilt resistance in brinjal) have motivated farmers to cultivate landraces. Similarly, unique biological traits such as—size in *Mituga* banana, colour^41^ in red rice *Kempu Sanna*, flavor^14^ in *Adderi Jeerige* (mango) and *Kothambri Saale* (rice) and/or specific use viz., pickles in case of mango^27^ and *Ibbudla* (cucumber) for making juice; preparation of traditional meals viz., sweet dish, puffed rice, *kaayi kadubu*, *subzi, patrode*^18,42^ also motivate farmers to conserve and promote conservation of landraces.

Many of the landraces are associated with the traditions and cultural practices of the communities. As a result, ethnic traditional cultural practices and customs play an important role in the preservation of traditional variations and crop genetic diversity on farms. Hence, conserving traditions indirectly helps to conserve landraces^43^. These motivations are in line with the results of Gajanana et al.^19^ in India and Alwis et al.^10^ in Sri Lanka. The present study is coherent with the results regarding the determinants/factors highlighted by Sthapit et al.^13^. Considering the high level of diversity among custodian farmers and improving their ties with other members of the community can result in on-farm agro-biodiversity conservation *in situ*^13,19^.

#### Exchange of conserved material

The seed exchange takes place between individuals or families inside the community or between close communities^12^. Seed flow occurs through purchase of seeds from inside or beyond the community mainly in *bio-diversity fairs* and *seed melas*, as well as seed borrowing from relatives and fellow farmers. These exchanges and borrowings occur in the study region for a variable number of reasons, including—lack of seed of a particular variety or landraces in the market; a desire to replace poor-quality seeds from old lots and seasons which may have poor germination; an interest in growing better cultivars by seeing other farmers' fields; a desire to test a different landrace/folk variety in search of suitable landrace to replace the existing one for specific land suitability; and exchanging seeds of one landrace for the seeds of different landrace. From the interaction, it was observed that, seed exchange among the farming communities is in practice for several years, which in turn increases the diversity in the farmers’ field and indirectly conserves the specific landrace. Normally, the custodian farmers have a practice of collecting and storing the seeds for the next growing season, contributing to the maintenance of the crop diversity. Custodian farmers are farmers (men and women) who actively maintain, adapt and promote agricultural biodiversity and related knowledge at farm and community levels over an extended period of time, and are recognized by community members for doing so^13^. Often, custodian farmers do not act alone, but rather are actively supported in their efforts by family or household members. These features of seed conservation and exchange were discussed by Conversa et al.^11^ in vegetable landraces conservation in the Puglia region of Italy. Similar kind of exchange was also found in our study area, where a collective exchange happens during events such as local markets or traditional ceremonies where a group of farmers or communities from different parts of the state exchange seeds through purchase or barter system. Furthermore, certain farmers in the study area had a great knowledge on the importance of conservation of landraces and they played an integrated role in motivating/encouraging/involving other fellow farmers in conservation. While interacting with a custodian farmer from Belagavi, who maintains a large number of landraces, we found that he distributed two to five landraces to interested farmers with a motive to increase area under landrace cultivation and to help the fellow farmers to sustain during difficult times. Thus, custodian farmers play a significant role in the seed flow and they are the main source of seeds in the region. Few farmers from Shivamogga develop nursery for sale of landraces, mainly pepper, jackfruit and pickling varieties of mango^36^. Through germplasm movement, these farmers are developing a dynamic process of diversity on their farms^22^. Increased cultivation of landraces achieved through seed exchange within and between communities, diversity fairs, and public awareness of the importance of landraces improves their use and conservation^34^.

### Total economic value

**Figure 7:**
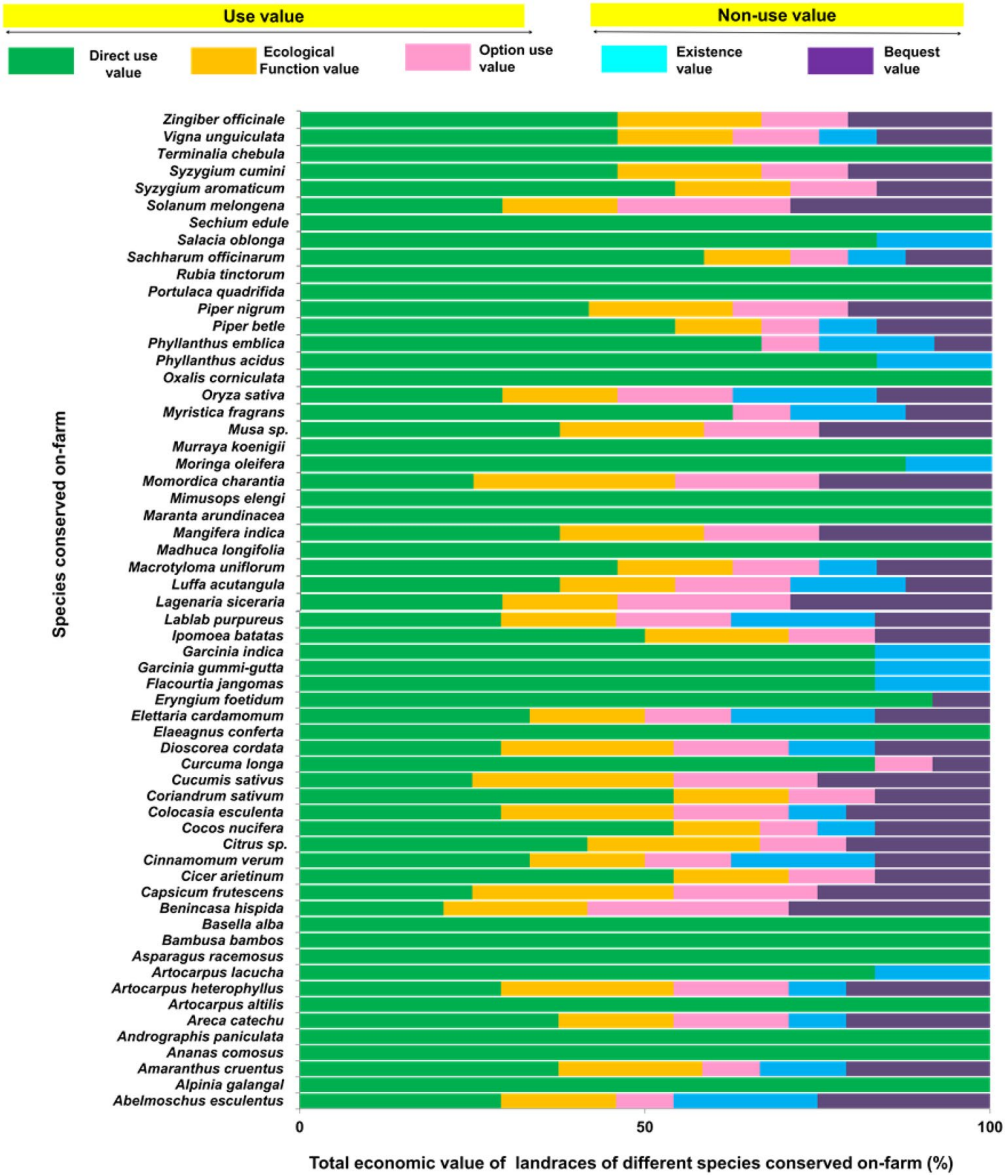
Total economic value of landraces of different species conserved on-farm by the farmers of Western Ghats.

Landraces are an essential component of agro-biodiversity conservation due to their direct and indirect benefits to farmers. The farmers conserve landraces to improve the sustainability of food, fuel, medical care and for future. Farmers value the landraces based on the importance. Poudel and Johnsen^44^ summarized the total economic value of crop landraces as inclusion of both use value (direct use value, ecological function value, and option use value) and non-use value (existence value and bequest value). Economic valuation of landrace diversity is essential to generate information and knowledge for resource allocation to identify least cost strategies to conserve landraces diversity^45^. Among several species of landraces conserved on-farm, 76.67% (46 species) landraces are conserved for both use and non-use values (Fig. 7). The landraces of the majority of the species (46) are conserved on-farm for either existence or/and bequest value in addition to direct use value, ecological function value and option value, highlights the farmers involvement in conservation of landraces for benefit of others in current and future generations.

### Constraints for conservation

The custodian farmers are doing their best to maintain, manage and promote the local varieties and landraces through seed exchange. In addition, they are passing traditional knowledge about these cultivars and disseminating their importance among their fellow farmers within and outside their community^13^. Unfortunately, the genetic diversity of landraces is rapidly diminishing in various parts of the world for a variety of reasons. This fact has also been supported by Hammer and Teklu^46^ citing the introduction of high yielding varieties (HYVs) leading to replacement of landraces/traditional folk varieties.

Conservation constraints are broadly divided into agro-ecological, socio-economic and technical aspects. Flooding, drought, rainfall during harvest, landslides, poor soil quality and abnormal weather are the main agro-ecological constraints. Whereas socio-economic constraints include inadequate input, lack of availability of seed material, poor yield, lack of marketing facilities, deterioration of culture and traditions, lack of awareness of conservation, adoption of HYVs, lack of interest among young people and their migration to urban areas, the non-multiplication of seeds by the family and poor knowledge transfer. Constraints of technical cultivation comprises of pest and disease infestation, labor scarcity, improper storage conditions and poor germination rate^14^. The Western Ghats encompass hilly area covered with forests that receive heavy rainfall deteriorating soil conditions, by erosion, flooding, submergence of fields, landslides that are common intimidations for cultivar conservation. Based on the information obtained from custodian farmers, the loss of many landraces over the years due to the frequent occurrence of natural calamities in the study area was highlighted. They also emphasized that, lack of prevalence with different farmers in many landraces, they are unable to protect those landraces. Socio-economic conditions make a huge impact on conservation and are the core threatening factors for the conservation of landraces. The practice of adoption of HYVs since the green revolution replaces several landraces, especially in Belagavi and Dakshina Kannada, due to the lack of seed availability and poor yield lead to decrease importance in production and maintenance, especially in rice. Lack of awareness is another major problem for local cultivar conservation, as only a handful of farmers are indulging in cultivar conservation in forest and hilly areas. An increase in desire for a luxurious life and other job opportunities with the inflow of money from natives residing in other cities, states, and countries; agriculture, which was formerly the main occupation of the people, has taken a backseat. In alignment with this, farms and fields in the survey area have been turned into residential plots and commercial (retail) buildings, abandoning cultivation and agriculture^47^.

Deterioration of culture and traditions in rural areas, poor knowledge transfer from elders, increasing technology in cultivation and commercialization of agriculture has led to reduced desire for conservation by young farmers and migration to urban areas^48^. There are no defined and proper market chains for landraces in the Western Ghats region, which also affects the cultivation. Alwis et al.^10^ discussed the marketing problems for tubers crops in Sri Lanka. The strengthening of international markets and export incentives for other products like HYVs and the commercialization of agriculture in the area results in further loss of landrace diversity in the future^48,49^.

The Western Ghats is a place of origin and diversity for many plants and animals including insects. Thus, a variety of pests and diseases attack on the plants are reported. Insect and non-insect pests like rodents, macaques, wild boars, peacocks and other birds are major threats during cropping and harvesting time^10^. Improper storage conditions lead to occurrence of storage pests and diseases, thereby enhancing the viability loss and poor germination^40,42^. Many of these threats and constraints can be overcome through proper strategies. Promoting self-interest and creating awareness on the importance of landraces would in turn boost the conservation, maintenance and cultivation of landraces.

## Conclusion and future implications

India has one of the top three genebanks in the world conserving more than 400K accessions of agri-horticultural crops. About 550 germplasm accessions of seed propagated crops belonging to the surveyed area are conserved in the genebank at ICAR-NBPGR. Our study adds specific information related to use including the indigenous technical knowledge to the passport data of these accessions. This addition is expected to enhance their immediate utilization. Furthermore, the number of landraces that are conserved on-farm in various indigenous crops across the vast swathes of the huge country remains inadequately documented. This report represents only a cross-sectional study of on-farm conservation in Central Western Ghats. Similar studies in other regions of the country need to be carried out to document landraces, their diversity and determinants of on-farm conservation practices.

Current report has documented three significant issues:

(i) On-Farm conservation is practiced by a very few custodian farmers. Younger generation appears to find no incentive to continue the conservation practices.
(ii) The on-farm conservation sites vary in size and crop-composition. Some landraces (particularly of rice) are conserved by multiple custodians signifying their culinary popularity.
(iii) Landraces being indivisible part of local cuisine and passion of custodian farmers are the most important reasons for con-farm conservation.

Possible ways to attract young farmers to on-farm conservation may include:

(i) Registration of landraces as farmers’ variety (wherever applicable) with PPV&FRA
(ii) Popularization of the landraces among niche urban customers may increase demand and sale-price.
(iii) Development of improved versions (agronomic value) of these landraces by breeders and researchers may open avenues of benefit sharing by custodian farmers.

With enhanced and assured income generation, next-generation farmers may find incentives to continue on-farm conservation. Else, weakening of cultural traditions, declining economic returns, and changing climate may lead to erosion and ultimately irreversible loss of these invaluable landraces.

## Data Availability

The data that were generated during the study as well as those that support the findings are included in the paper. The data are also accessible from a database (under development and unpublished) at http://pgrinformatics.nbpgr.ernet.in/onfc/database.aspx.
